# Impact of adjuvant immunotherapy on prognosis in esophageal squamous cell carcinoma patients following neoadjuvant immunochemotherapy

**DOI:** 10.3389/fonc.2026.1735049

**Published:** 2026-03-18

**Authors:** Jiayi Geng, Bengang Hui, Teng Mu, Ni Ping, Runmin Jiang, Xiuyuan Chen, Heng Zhao, Dan zeng duo ji, Xizhao Sui, Yun Li, Xun Wang, Jie Lei

**Affiliations:** 1 Department of Thoracic Surgery, Peking University People’s Hospital, Beijing, China; 2 Thoracic Oncology Institute, Peking University People’s Hospital, Beijing, China; 3 Research Unit of Intelligence Diagnosis and Treatment in Early Non-small Cell Lung Cancer, Chinese Academy of Medical Sciences, Peking University People’s Hospital, Beijing, China; 4 Institute of Advanced Clinical Medicine, Peking University, Beijing, China; 5 Beijing Key Laboratory of Innovative Application of Big Data in Lung Cancer, Peking University People’s Hospital, Beijing, China; 6 Department of Thoracic Surgery, Tangdu Hospital, the Fourth Military Medical University, Xi’an, Shaanxi, China; 7 Department of Thoracic Surgery, The First Affiliated Hospital of Zhengzhou University, Zhengzhou, China; 8 Department of Cardio-thoracic Surgery, The Tibet Autonomous Region People’s Hospital, Lhasa, China

**Keywords:** adjuvant therapy, esophageal squamous cell carcinoma, immunochemotherapy, immunotherapy, neoadjuvant therapy

## Abstract

**Introduction:**

The CheckMate-577 trial confirmed that adjuvant nivolumab significantly prolonged disease-free survival (DFS) in patients with esophageal cancer who had residual disease after neoadjuvant chemoradiotherapy (nCRT). However, whether postoperative adjuvant immunotherapy (AIT) is necessary following neoadjuvant immunochemotherapy (nICT) remains highly controversial. This study aims to explore the efficacy of AIT in esophageal squamous cell carcinoma (ESCC) patients who underwent nICT.

**Methods:**

A multicenter, retrospective study was conducted on 323 ESCC patients who underwent nICT followed by R0 resection. Patients were divided into AIT group and non-AT group, with further stratification based on pCR status. Stable inverse probability of treatment weighting (sIPTW) was used to balance baseline characteristics. Primary endpoint was overall survival (OS). Secondary endpoints were disease-free survival (DFS) and cancer-specific survival (CSS). Survival outcomes were analyzed using Kaplan-Meier curves.

**Results:**

In overall patients, AIT significantly improved OS (2-year OS: 95.8% vs. 84.4%, P = 0.008) and CSS (2-year CSS: 96.8% vs. 89.9%, P = 0.036), while no significant survival benefit from AIT was observed for pCR patients. However, in non-pCR patients, AIT significantly improved OS (2-year OS: 93.5% vs. 78.8%, P = 0.024) and CSS (2-year CSS: 95.0% vs. 85.9%, P = 0.047). No significant differences were observed between adjuvant immunochemotherapy (AICT) and adjuvant immune checkpoint inhibitor monotherapy (AIMT).

**Conclusion:**

The study showed that AIT could bring prognosis benefits for patients receiving nICT, especially in non-pCR patients.

## Introduction

Neoadjuvant therapy followed by surgery is the standard approach for locally advanced esophageal squamous cell carcinoma (LA-ESCC). However, recurrence risks persist postoperatively, and survival outcomes remain suboptimal. Previous studies have demonstrated that adjuvant chemotherapy after nCRT improves prognosis in these patients (1). The CheckMate-577 trial further confirmed that adjuvant nivolumab significantly prolonged DFS in patients with residual disease after nCRT (2). Based on the findings of CheckMate-577 study, AIT has become a first-line recommendation for postoperative adjuvant treatment in patients with residual tumors following neoadjuvant chemoradiotherapy or chemotherapy.

With the increasing integration of immunotherapy into earlier treatment stages, nICT has emerged as a primary strategy for LA-ESCC in China. Multiple studies have validated the efficacy and safety of nICT. For instance, the NICE trial reported 2-year OS and recurrence-free survival (RFS) rates of 78.1% and 67.9%, respectively (3), while the Keystone-001 trial demonstrated even higher 2-year OS and DFS rates of 91% and 89% (4). Nevertheless, limited research has focused on adjuvant therapy following nICT. In most phase II trials, such as the NICE study, postoperative adjuvant therapy was administered at the discretion of treating physicians, whereas others, like the Keynote-001 trial, predominantly adopted adjuvant immunotherapy. Notably, two ongoing phase III trials, ESCORT-NEO/NCCES01 and HCHTOG1909, have adopted a perioperative approach combining neoadjuvant therapy, surgery, and adjuvant immunotherapy (5, 6).

Whether postoperative adjuvant immunotherapy is necessary following nICT remains highly controversial, given the potential long-term effects of immunotherapy. Current retrospective studies have yielded inconsistent conclusions. To address this gap, we conducted this multicenter, large-sample retrospective study to evaluate whether adjuvant immunotherapy provides survival benefits for LA-ESCC patients following nICT.

## Materials and methods

### Study population

This study retrospectively enrolled LA-ESCC patients who underwent nICT followed by surgical resection between January 2020 and December 2023 at the Fourth Military Medical University Tangdu Hospital, Peking University People’s Hospital, the First Affiliated Hospital of Zhengzhou University, and the People’s Hospital of Tibet Autonomous Region.

Inclusion criteria were as follows (1): Pathological diagnosis of ESCC via endoscopy before treatment, with clinical staging of cT2-4aNanyM0 confirmed by PET-CT, endoscopic ultrasound, contrast-enhanced CT, or non-contrast CT (2); Age≥18 years (3); Receipt of 1–4 cycles of NICT (4); Undergoing radical esophagectomy with postoperative pathological confirmation of R0 resection (5); Survival for more than 90 days postoperatively (6); Receipt of AIT within 3 months after surgery.

Exclusion criteria were (1): Pathological diagnosis of esophageal adenocarcinoma or other types of esophageal cancer (2); Palliative surgery or failure to achieve R0 resection (3); Receipt of radiotherapy during neoadjuvant or adjuvant treatment (4); Concurrent malignancies at other sites (5); Severe missing clinical or follow-up data.

### Study endpoints

The primary endpoint of this study was OS. The second endpoints included DFS, CSS and patterns of recurrence and metastasis. In terms of recurrence and metastasis patterns, regional recurrence was defined as recurrence at the tumor bed, anastomotic site, or regional lymph nodes while distant metastasis was defined as metastasis to other organs, pleura, peritoneum, or non-regional lymph nodes.

### Adjuvant immunotherapy

AIT was defined as any postoperative adjuvant therapy involving immune checkpoint inhibitors (ICIs) initiated within 3 months after radical surgery. AIT was not mandatory, and patients who received at least one cycle of adjuvant treatment were considered to have undergone adjuvant therapy. In this study, AIT regimens included adjuvant immune checkpoint inhibitors monotherapy (AIMT) and adjuvant immunochemotherapy (AICT). The specific regimen and duration of AIT were determined by the treating physician based on the patient’s condition, with adjustments made according to patient tolerance, and tumor response.

### Follow-up

Patients were followed up every 3–6 months during the first 2 years after surgery, every 6 months during the 3rd to 5th years and annually thereafter. Follow-up information was obtained through outpatient records and telephone interviews.

### Statistical analysis

Statistical analysis was performed using R 4.4.2. Kaplan-Meier curves and log-rank tests were used to analyze survival differences. The t-test was used for normally distributed variables, while the Mann-Whitney U test was used for non-normally distributed continuous variables. Categorical variables were expressed as frequencies and percentages, and comparisons were made using Pearson’s chi-square test or Fisher’s exact test. Since postoperative adjuvant therapy was not randomized, stable inverse probability of treatment weighting (sIPTW) was used to adjust for differences between the two groups. Propensity scores (PS) were calculated using a multivariate logistic regression model, and baseline characteristics with a standardized mean difference (SMD)≤0.1 were considered balanced. The sIPTW for each patient was calculated as follows: for the adjuvant therapy group, Pt/PS, where Pt represents the proportion receiving AIT; for the non-adjuvant therapy group, (1-Pt)/(1-PS). A two-sided P<0.05 was considered statistically significant.

## Results

### Baseline characteristics and pathologic outcomes

**Figure 1:**
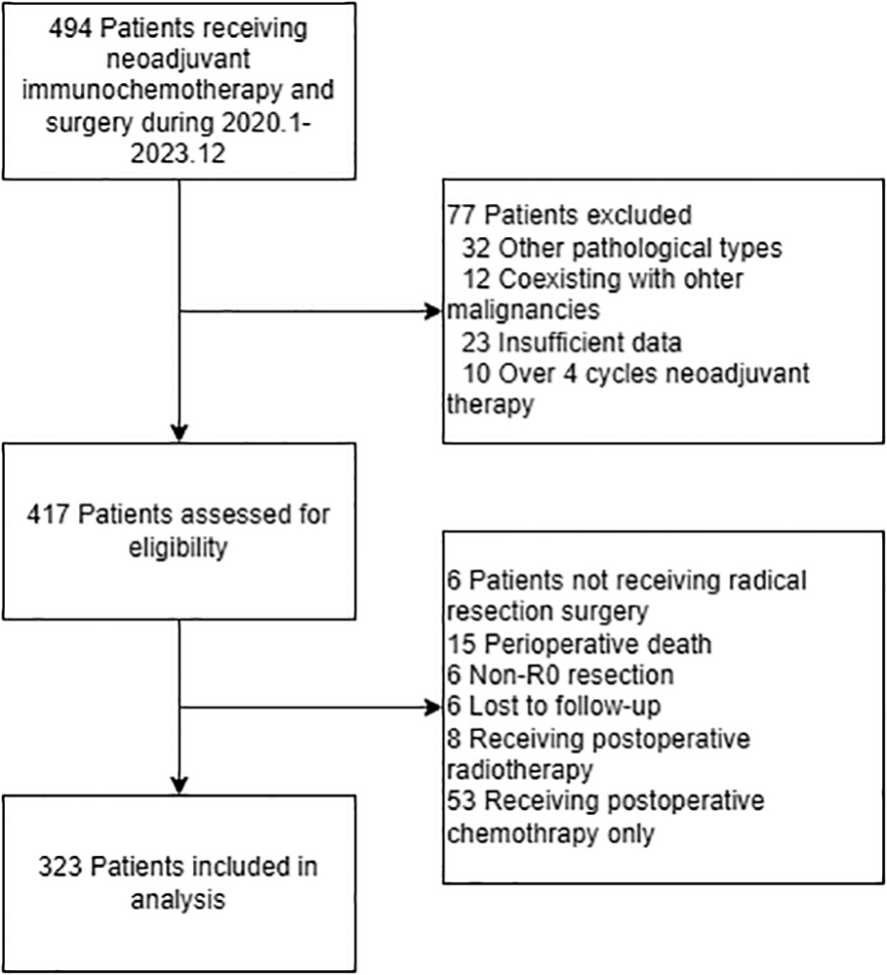
Flowchart of the study.

A total of 323 patients were ultimately included in the study (**Figure 1**), of whom 163 (50.5%) received postoperative AIT, while 160 (49.5%) did not receive postoperative adjuvant therapy (non-AT). The baseline characteristics and postoperative pathologic information of the patients are presented in **Table 1**. Patients who received AIT had a higher proportion of tumors located in the upper thoracic segment, more advanced clinical staging, a higher proportion of receiving 3–4 cycles of neoadjuvant therapy, a slightly lower proportion of using TP/DP regimens during neoadjuvant chemotherapy, a slightly higher proportion of using PD-1 inhibitors, more advanced postoperative pathological staging, and poorer pathological responses (SMD > 0.1). After sIPTW, the baseline characteristics of the patients were well-balanced. Among the patients who received postoperative adjuvant therapy, 107 (65.6%) underwent AICT, while 56 (34.4%) received AIMT. The specific regimens of adjuvant chemotherapy administered to the patients are detailed in **Supplementary Table 1**.

**Table 1:** Baseline characteristics and pathologic outcomes.

Characteristic	Before sIPTW				After sIPTW			
	AIT	Non-AT	SMD	P	AIT	Non-AT	SMD	P
Number	163	160			162.4	155.9		
Sex (n, %)			0.023	0.27			0.003	0.98
Male	127 (77.9)	118 (73.8)			123.1 (75.8)	118.0 (75.7)		
Female	60 (36.8)	42 (26.2)			39.3 (24.2)	37.9 (24.3)		
Age (n, %)			0.097	0.46			0.016	0.90
≥65 years	60 (36.8)	80 (50.0)			71.0 (43.7)	86.5 (55.5)		
<65 years	103 (63.2)	80 (50.0)			91.3 (56.3)	69.4 (44.5)		
Smoking history (n, %)	85 (52.1)	77 (48.1)	0.081	0.54	85.2 (52.5)	80.8 (51.8)	0.013	0.92
Drinking history (n, %)	62 (38.8)	58 (35.6)	0.066	0.64	63.2 (38.9)	60.3 (38.7)	0.005	0.97
Location (n, %)			0.238	0.11			0.055	0.91
Upper	9 (5.5)	2 (1.2)			5.5 (3.4)	3.9 (2.5)		
Middle	55 (33.7)	56 (35.0)			57.7 (35.5)	54.6 (35.0)		
Lower	99 (60.7)	102 (63.7)			99.2 (61.1)	97.4 (62.5)		
Clinical stage (n, %)			0.193	0.23			0.034	0.96
II	66 (40.5)	69 (43.1)			70.6 (43.5)	66.9 (42.9)		
III	82 (50.3)	84 (52.5)			81.4 (50.1)	80.2 (51.4)		
IV	15 (9.2)	7 (4.4)			10.4 (6.4)	8.9 (5.7)		
Neoadjuvant chemotherapy regimen (n, %)			0.11	0.52			0.016	0.89
TP/DP	157 (97.5)	157 (98.1)			158.1 (97.4)	3.7 (2.4)		
CF	6 (3.7)	3 (1.9)			4.3 (2.6)	152.2 (97.6)		
Neoadjuvant ICIs (n, %)			0.24	0.11			0.043	0.70
PD-1	159 (97.5)	152 (95.0)			157.9 (97.3)	5.4 (3.5)		
PD-L1	4 (2.5)	8 (5.0)			4.4 (2.7)	150.5 (96.5)		
Neoadjuvant cycles (n, %)			0.29	0.014			0.018	0.88
1-2	104 (63.8)	123 (76.9)			114.9 (70.8)	44.3 (28.4)		
3-4	59 (36.2)	37 (23.1)			47.5 (29.2)	111.6 (71.6)		
ypT (n, %)			0.38	0.05			0.091	0.98
T0	42 (25.8)	66 (41.2)			55.2 (34.0)	53.9 (34.6)		
Tis	10 (6.1)	4 (2.5)			7.1 (4.4)	5.4 (3.5)		
T1	40 (24.5)	35 (21.9)			38.2 (23.5)	37.4 (24.0)		
T2	21 (12.9)	19 (11.9)			20.7 (12.8)	19.9 12.8)		
T3	47 (28.8)	35 (11.9)			39.7 (24.4)	38.8 (24.9)		
T4	3 (1.8)	1 (0.6)			1.5 (0.9)	0.5 (0.3)		
ypN (n, %)			0.33	0.037			0.052	0.98
N0	107 (65.6)	123 (76.9)			117.2 (72.2)	112.7 (72.3)		
N1	44 (27.0)	24 (15.0)			34.9 (21.5)	33.1 (21.2)		
N2	10 (6.1)	8 (5.0)			8.2 (5.1)	7.3 (4.7)		
N3	2 (1.2)	5 (3.1)			2.0 (1.2)	2.9 (1.8)		
ypTNM (n, %)			0.36	0.018			0.03	>0.99
I	84 (51.5)	107 (66.9)			97.2 (59.9)	94.3 (60.5)		
II	21 (12.9)	16 (10.0)			19.0 (11.7)	18.4 (11.8)		
III	54 (33.1)	31 (19.4)			43.2 (26.6)	39.8 (25.5)		
IV	4 (2.5)	6 (3.8)			3.0 (1.9)	3.4 (2.2)		
Pathological response (n, %)			0.36	0.007			0.005	>0.99
pCR	36 (22.1)	60 (37.5)			49.0 (30.2)	47.5 (30.4)		
MPR	38 (23.3)	25 (15.6)			31.2 (19.2)	29.7 (19.1)		
IPR	89 (54.6)	75 (46.9)			82.1 (50.6)	78.7 (50.5)		

Considering the significant prognostic differences between patients achieving pCR and non-pCR, we performed stratified analyses based on pathologic response status. The detailed baseline characteristics and pathologic outcomes of pCR and non-pCR patients were presented in **Table 2**, **3**.

**Table 2:** Baseline characteristics of pCR patients.

Characteristic	Before sIPTW				After sIPTW			
	AIT	Non-AT	SMD	P	AIT	Non-AT	SMD	P
Number	36	60			35.6	59.2		
Sex (n, %)			0.11	0.77			0.017	0.94
Male	24 (66.7)	43 (71.7)			25.5 (71.5)	41.8 (70.7)		
Female	12 (33.3)	17 (28.3)			10.2 (28.5)	17.3 (29.3)		
Age (n, %)			0.39	0.11			0.007	0.98
≥65 years	23 (63.9)	27 (45.0)			17.6 (49.3)	29.0 (49.0)		
<65 years	13 (36.1)	33 (55.0)			18.1 (50.7)	30.2 (51.0)		
Smoking history (n, %)	16 (44.4)	26 (43.3)	0.022	>0.99	16.3 (45.8)	26.1 (44.1)	0.035	0.88
Drinking history (n, %)	8 (22.2)	19 (31.7)	0.052	0.81	11.2 (31.5)	17.6 (29.8)	0.037	0.88
Location (n, %)			0.17	0.73			0.035	>0.99
Upper	1 (2.8)	1 (1.7)			0.6 (1.8)	1.1 (1.8)		
Middle	15 (41.7)	21 (35.0)			12.6 (35.4)	21.9 (37.0)		
Lower	20 (55.6)	38 (63.3)			22.4 (62.8)	36.2 (61.1)		
Clinical stage (n, %)			0.18	0.69			0.025	>0.99
II	21 (58.3)	30 (50.0)			18.4 (51.7)	31.2 (52.7)		
III	13 (36.1)	27 (45.0)			15.6 (43.7)	25.1 (42.4)		
IV	2 (5.6)	3 (5.0)			1.6 (4.6)	2.9 (4.8)		
Neoadjuvant chemotherapy regimen (n, %)			0.075	>0.99			<0.001	>0.99
TP/DP	35 (97.2)	59 (93.3)			35.1 (98.4)	58.3 (98.4)		
CF	1 (2.8)	1 (1.7)			0.6 (1.6)	0.9 (1.6)		
Neoadjuvant ICIs (n, %)			0.38	0.29			0.13	0.52
PD-1	36 (100.0)	56 (93.3)			35.6 (100.0)	56.7 (95.8)		
PD-L1	0 (0)	4 (6.7)			0 (0.0)	2.5 (4.2)		
Neoadjuvant cycles (n, %)			0.36	0.14			0.015	0.95
1-2	15 (41.7)	15 (25.0)			11.1 (31.1)	18.0 (30.4)		
3-4	21 (58.3)	45 (75.0)			24.6 (68.9)	41.2 (69.6)		

**Table 3:** Baseline characteristics and pathologic outcomes of non-pCR patients.

Characteristic	Before sIPTW				After sIPTW			
	AIT	Non-AT	SMD	P	AIT	Non-AT	SMD	P
Number	127	100			125.7	97.6		
Sex (n, %)			0.15	0.34			<0.001	>0.99
Male	103 (81.1)	75 (75.0)			100.2 (79.7)	77.8 (79.7)		
Female	24 (18.9)	25 (25.0)			25.5 (20.3)	19.8(20.3)		
Age (n, %)			0.20	0.17			0.017	0.90
≥65 years	47 (37.0)	47 (47.0)			52.8 (42.0)	40.2 (41.1)		
<65 years	80 (63.0)	53 (53.0)			72.9 (58.0)	57 (58.9)		
Smoking history (n, %)	69 (54.3)	51 (51.0)	0.067	0.72	70.5 (56.1)	54.5 (55.8)	0.005	0.97
Drinking history (n, %)	50 (39.4)	43 (43.0)	0.074	0.68	54.4 (43.3)	42.1 (43.1)	0.004	0.98
Location (n, %)			0.29	0.12			0.028	0.98
Upper	8 (6.3)	1 (1.0)			5.1 (4.0)	3.4 (3.5)		
Middle	40 (31.5)	35 (35.0)			42.6 (33.9)	33.5 (34.4)		
Lower	79 (62.2)	64 (64.0)			78.0 (62.1)	60.6 (62.1)		
Clinical stage (n, %)			0.25	0.21			0.052	0.94
II	45 (35.4)	39 (39.0)			47.5 (37.7)	35.4 (36.2)		
III	69 (54.3)	57 (57.0)			69.1 (55.0)	56.0 (57.3)		
IV	13 (10.2)	4 (4.0)			9.1 (7.3)	6.3 (6.4)		
Neoadjuvant chemotherapy regimen (n, %)			0.11	0.65			0.024	0.87
TP/DP	122 (96.1)	96 (96.0)			3.8 (3.0)	2.6 (2.6)		
CF	5 (3.9)	4 (4.0)			121.9 (97.0)	95.0 (97.4)		
Neoadjuvant ICIs (n, %)			0.046	>0.99			0.002	0.99
PD-1	123 (96.9)	98 (98.0)			121.9 (96.9)	94.6 (96.9)		
PD-L1	4 (3.1)	2 (2.0)			3.9 (3.1)	3.0 (3.1)		
Neoadjuvant cycles (n, %)			0.28	0.05			0.033	0.83
1-2	83 (65.4)	78 (78.0)			89.0 (70.8)	70.5 (72.3)		
3-4	44 (34.6)	22 (22.0)			36.7 (29.2)	27.1 (27.7)		
ypT (n, %)			0.22	0.77			0.10	0.98
T0	7 (5.5)	6 (6.0)			7.9 (6.3)	7.0 (7.1)		
Tis	10 (7.9)	4 (4.0)			8.0 (6.4)	4.9 (5.0)		
T1	39 (30.7)	35 (35.0)			41.1 (32.7)	33.6 (34.5)		
T2	21 (16.5)	19 (19.0)			22.7 (18.1)	16.8 (17.2)		
T3	47 (37.0)	35 (35.0)			44.4 (35.3)	34.9 (35.7)		
T4	3 (2.4)	1 (1.0)			1.7 (1.3)	0.4 (0.5)		
ypN (n, %)			0.29	0.19			0.054	>0.99
N0	71 (55.9)	63 (63.0)			75.8 (60.3)	59.6 (61.1)		
N1	44 (34.6)	24 (24.0)			38.1 (30.3)	28.9 (29.6)		
N2	10 (7.9)	8 (8.0)			9.3 (7.4)	6.5 (6.7)		
N3	2 (1.6)	5 (5.0)			2.5 (2.0)	2.6 (2.6)		
ypTNM (n, %)			0.28	0.25			0.042	>0.99
I	48 (37.8)	47 (47.0)			53.7 (42.7)	43.3 (44.4)		
II	21 (16.5)	16 (16.0)			21.0 (16.7)	16.3 (16.7)		
III	54 (42.5)	31 (31.0)			47.4 (37.7)	35.0 (35.8)		
IV	4 (3.1)	6 (6.0)			3.6 (2.9)	3.0 (3.1)		
Pathological response (n, %)			0.11	0.50			0.011	0.94
MPR	89 (70.1)	75 (75.0)			91.7 (72.9)	70.7 (72.4)		
IPR	38 (29.9)	25 (25.0)			34.1 (27.1)	26.9 (27.6)		

### Survival analysis

**Figure 2:**
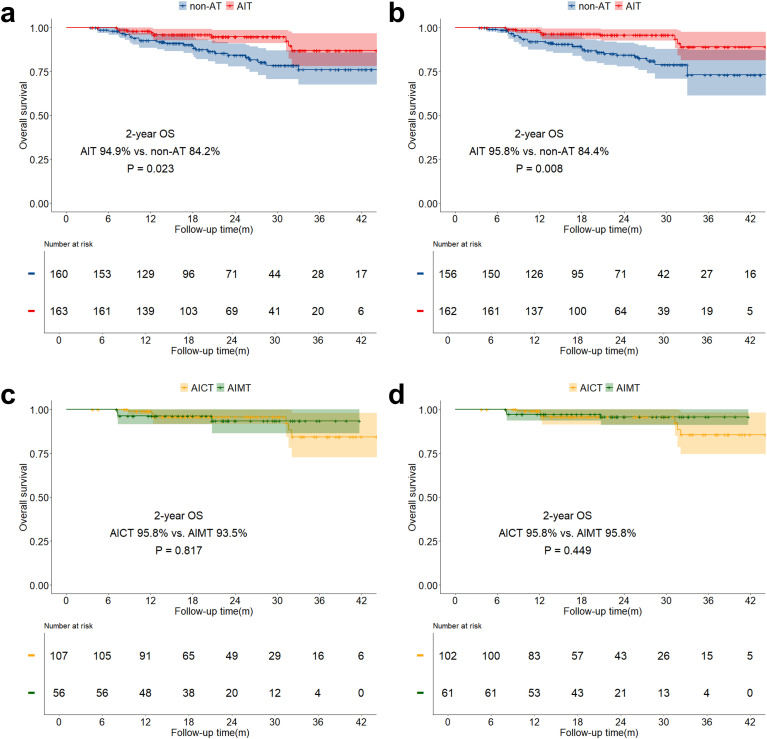
OS curves before and after sIPTW for the entire population, stratified by adjuvant therapy status. **(a)** Comparison of OS between the AIT group and the non-AT group before sIPTW; **(b)** Comparison of OS between the AIT group and the non-AT group after sIPTW; **(c)** Comparison of OS between the AICT group and the AIMT group before sIPTW; **(d)** Comparison of OS between the AICT group and the AIMT group after sIPTW.

**Figure 3:**
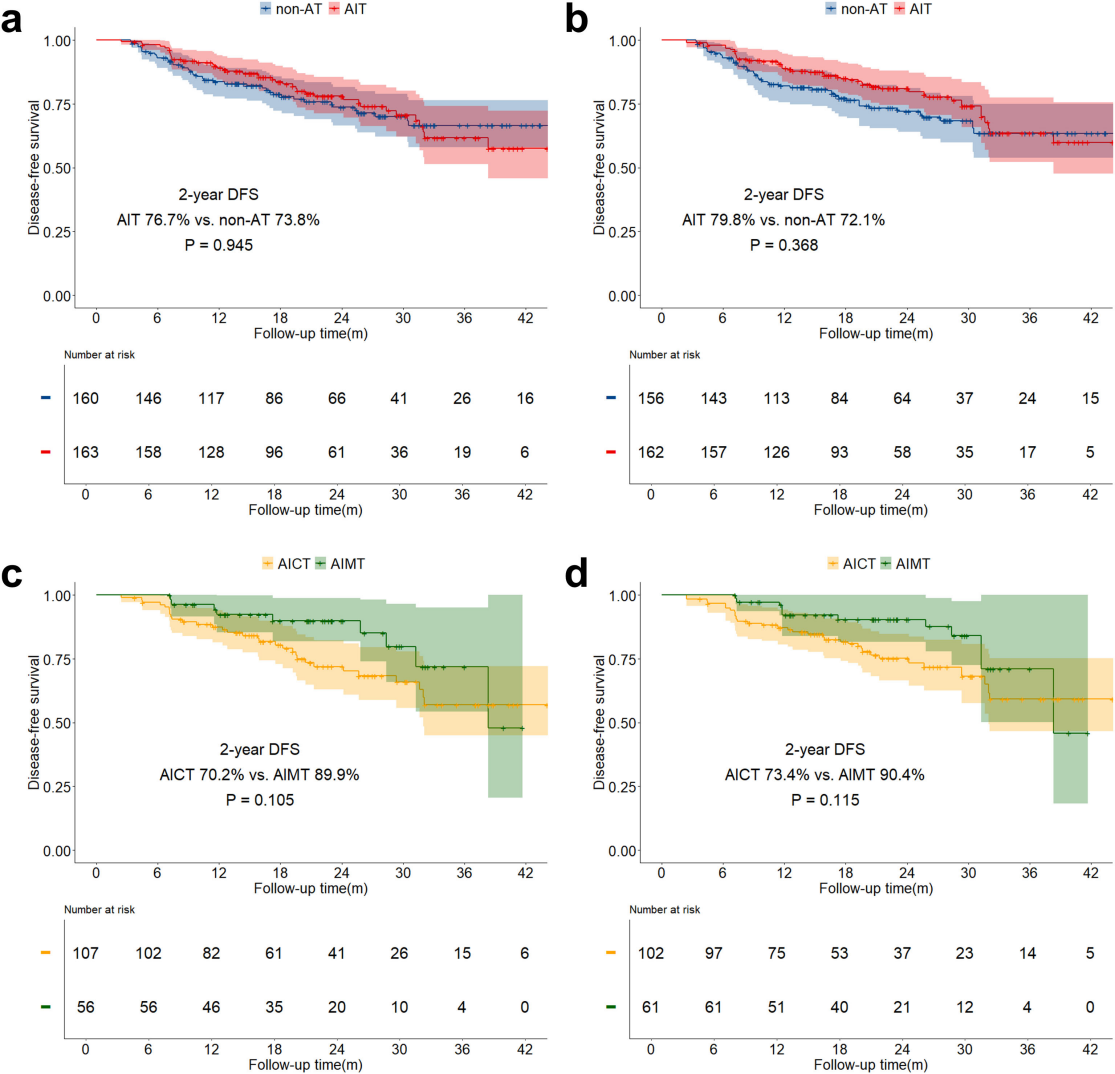
DFS curves before and after sIPTW for the entire population, stratified by adjuvant therapy status. **(a)** Comparison of DFS between the AIT group and the non-AT group before sIPTW; **(b)** Comparison of DFS between the AIT group and the non-AT group after sIPTW; **(c)** Comparison of DFS between the AICT group and the AIMT group before sIPTW; **(d)** Comparison of DFS between the AICT group and the AIMT group after sIPTW.

**Figure 4:**
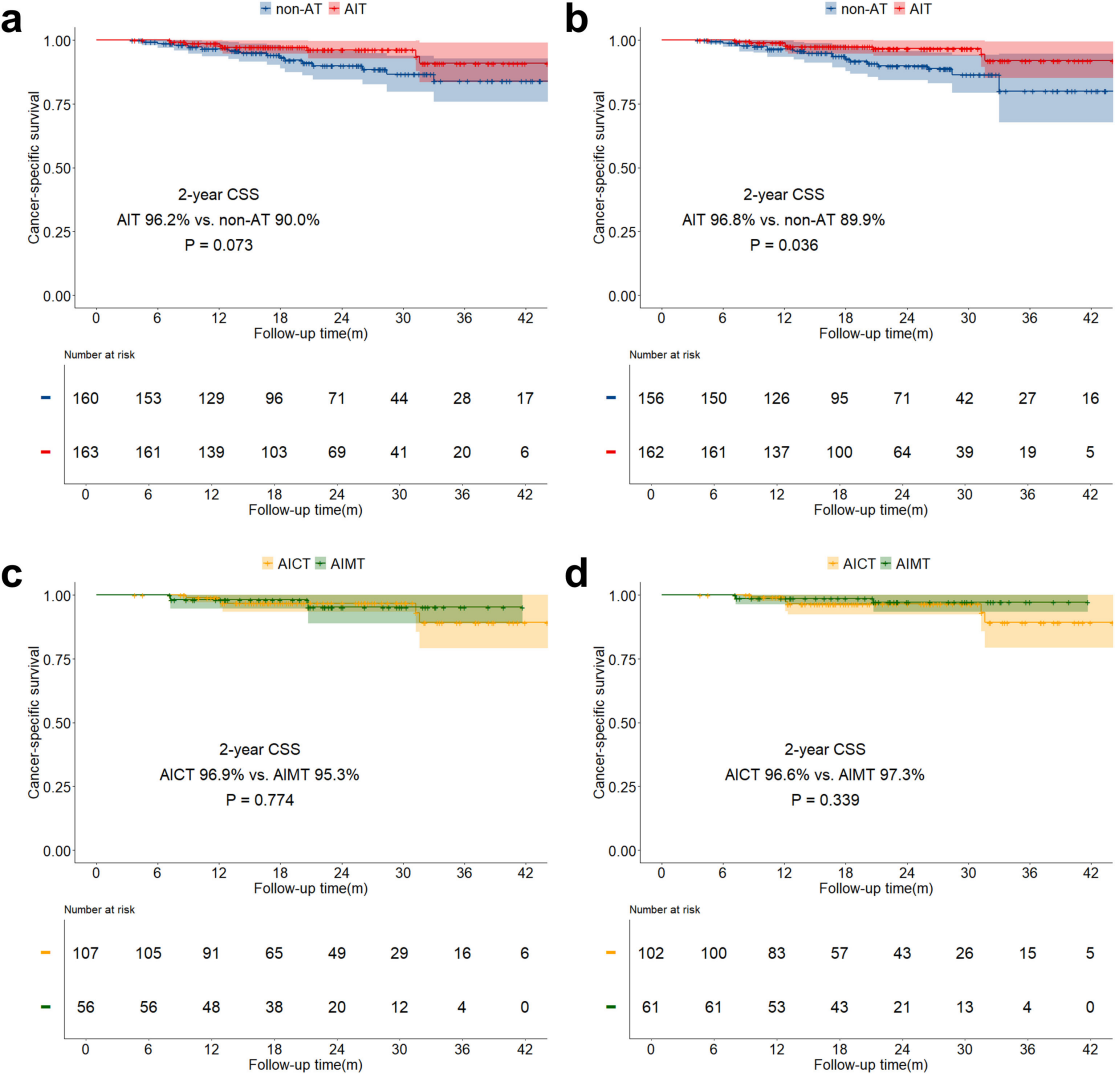
CSS curves before and after sIPTW for the entire population, stratified by adjuvant therapy status. **(a)** Comparison of CSS between the AIT group and the non-AT group before sIPTW; **(b)** Comparison of CSS between the AIT group and the non-AT group after sIPTW; **(c)** Comparison of CSS between the AICT group and the AIMT group before sIPTW; **(d)** Comparison of CSS between the AICT group and the AIMT group after sIPTW.

The median follow-up time for the study was 21.6 months (IQR: 14.0 months - 30.4 months). The overall two-year OS was 89.6% (IQR: 85.8% - 93.5%), and the two-year DFS was 75.2% (IQR: 70.1% - 80.8%). Both before and after sIPTW, the 2-year OS in the AIT group was significantly better than that in the non-AT group (before sIPTW: 94.9% vs. 84.2%, P = 0.023; after sIPTW: 95.8% vs. 84.4%, P = 0.008; **Figures 2a, b**). There was no significant difference in OS between the AICT group and the AIMT group (**Figures 2c, d**). Before and after sIPTW, there were no statistical differences in 2-year DFS between the AIT group and the non-AT group (before sIPTW: 76.7% vs. 73.8%, P = 0.945; after sIPTW: 79.8% vs. 72.1%, P = 0.368; **Figures 3a, b**). Different adjuvant immunotherapy modalities also did not show differences in DFS (**Figures 3c, d**). After sIPTW, the AIT group demonstrated a benefit in 2-year CSS (before sIPTW: 96.2% vs. 90.0%, P = 0.073; after sIPTW: 96.8% vs. 89.9%, P = 0.036; **Figures 4a, b**), while the AICT group and the AIMT group did not show differences in CSS (**Figures 4c, d**).

**Figure 5:**
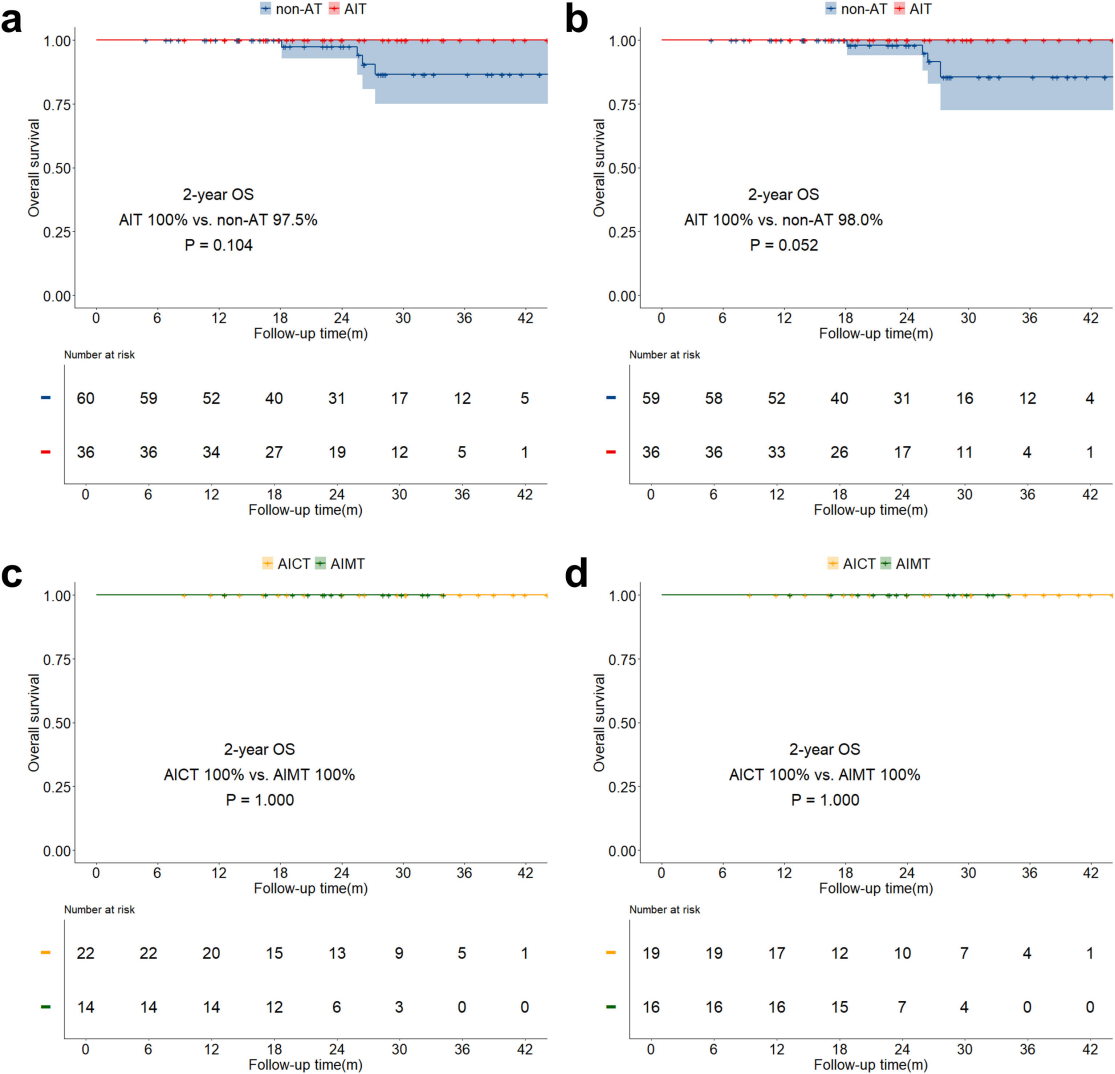
OS curves before and after sIPTW for patients achieving pCR, stratified by adjuvant therapy status. **(a)** Comparison of OS between the AIT group and the non-AT group before sIPTW; **(b)** Comparison of OS between the AIT group and the non-AT group after sIPTW; **(c)** Comparison of OS between the AICT group and the AIMT group before sIPTW; **(d)** Comparison of OS between the AICT group and the AIMT group after sIPTW.

**Figure 6:**
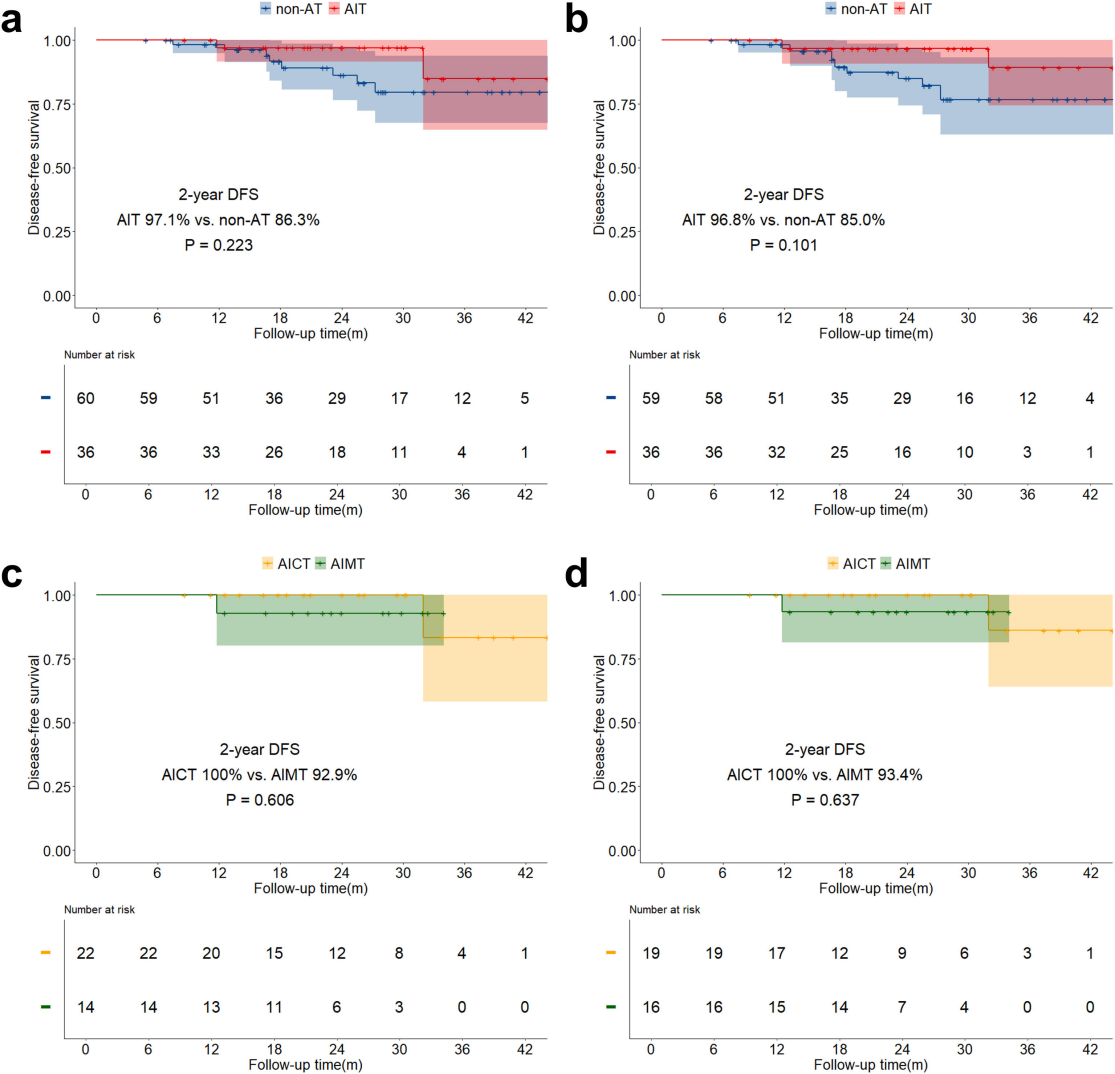
DFS curves before and after sIPTW for patients achieving pCR, stratified by adjuvant therapy status. **(a)** Comparison of DFS between the AIT group and the non-AT group before sIPTW; **(b)** Comparison of DFS between the AIT group and the non-AT group after sIPTW; **(c)** Comparison of DFS between the AICT group and the AIMT group before sIPTW; **(d)** Comparison of DFS between the AICT group and the AIMT group after sIPTW.

**Figure 7:**
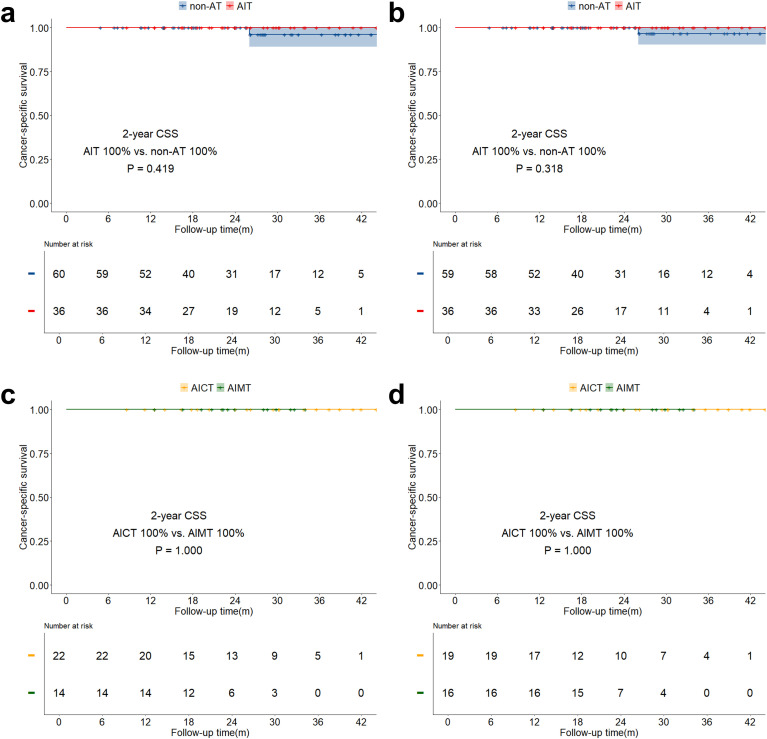
CSS curves before and after sIPTW for patients achieving pCR, stratified by adjuvant therapy status. **(a)** Comparison of CSS between the AIT group and the non-AT group before sIPTW; **(b)** Comparison of CSS between the AIT group and the non-AT group after sIPTW; **(c)** Comparison of CSS between the AICT group and the AIMT group before sIPTW; **(d)** Comparison of CSS between the AICT group and the AIMT group after sIPTW.

**Figure 8:**
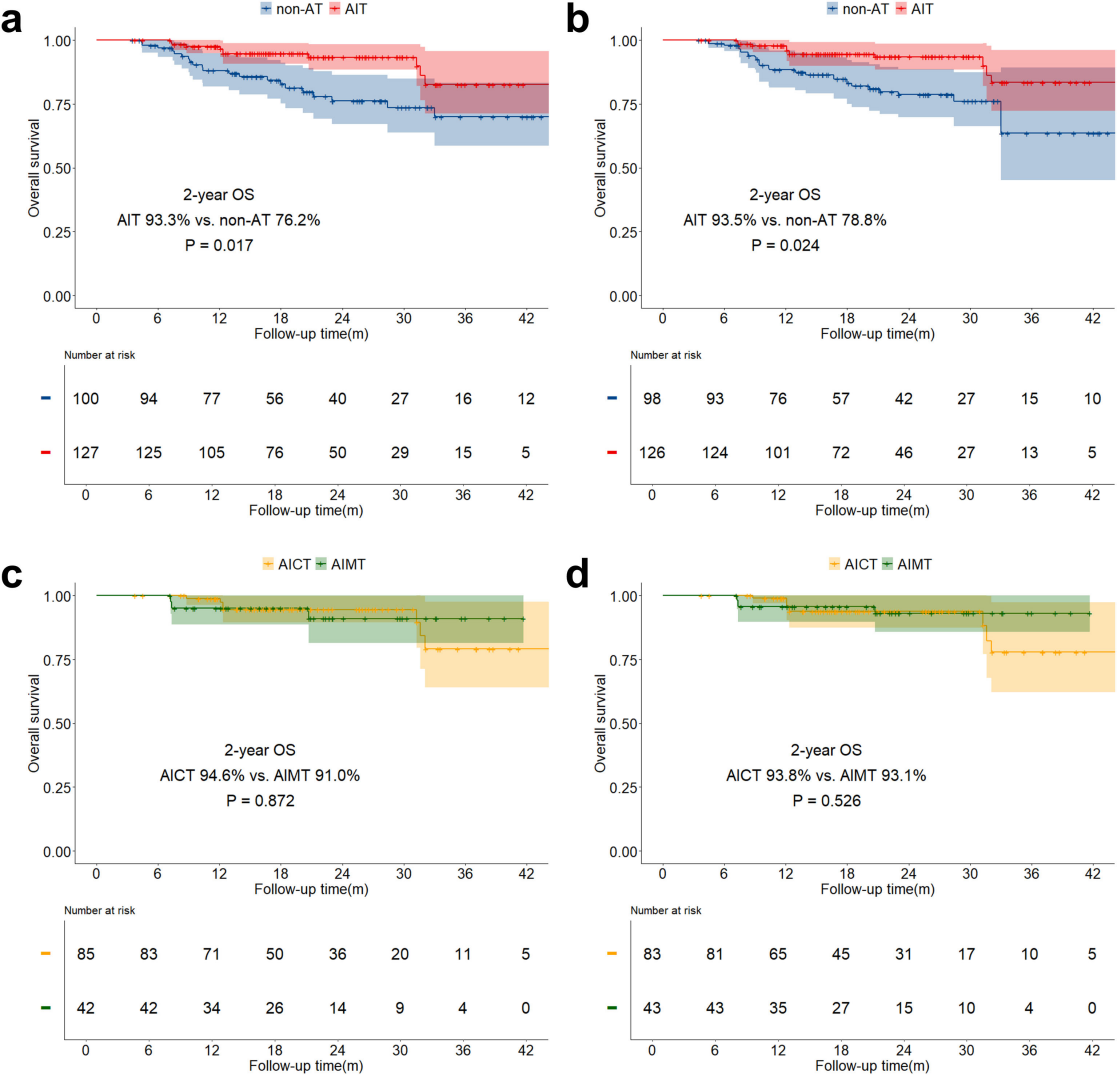
OS curves before and after sIPTW for non-pCR patients, stratified by adjuvant therapy status. **(a)** Comparison of OS between the AIT group and the non-AT group before sIPTW; **(b)** Comparison of OS between the AIT group and the non-AT group after sIPTW; **(c)** Comparison of OS between the AICT group and the AIMT group before sIPTW; **(d)** Comparison of OS between the AICT group and the AIMT group after sIPTW.

**Figure 9:**
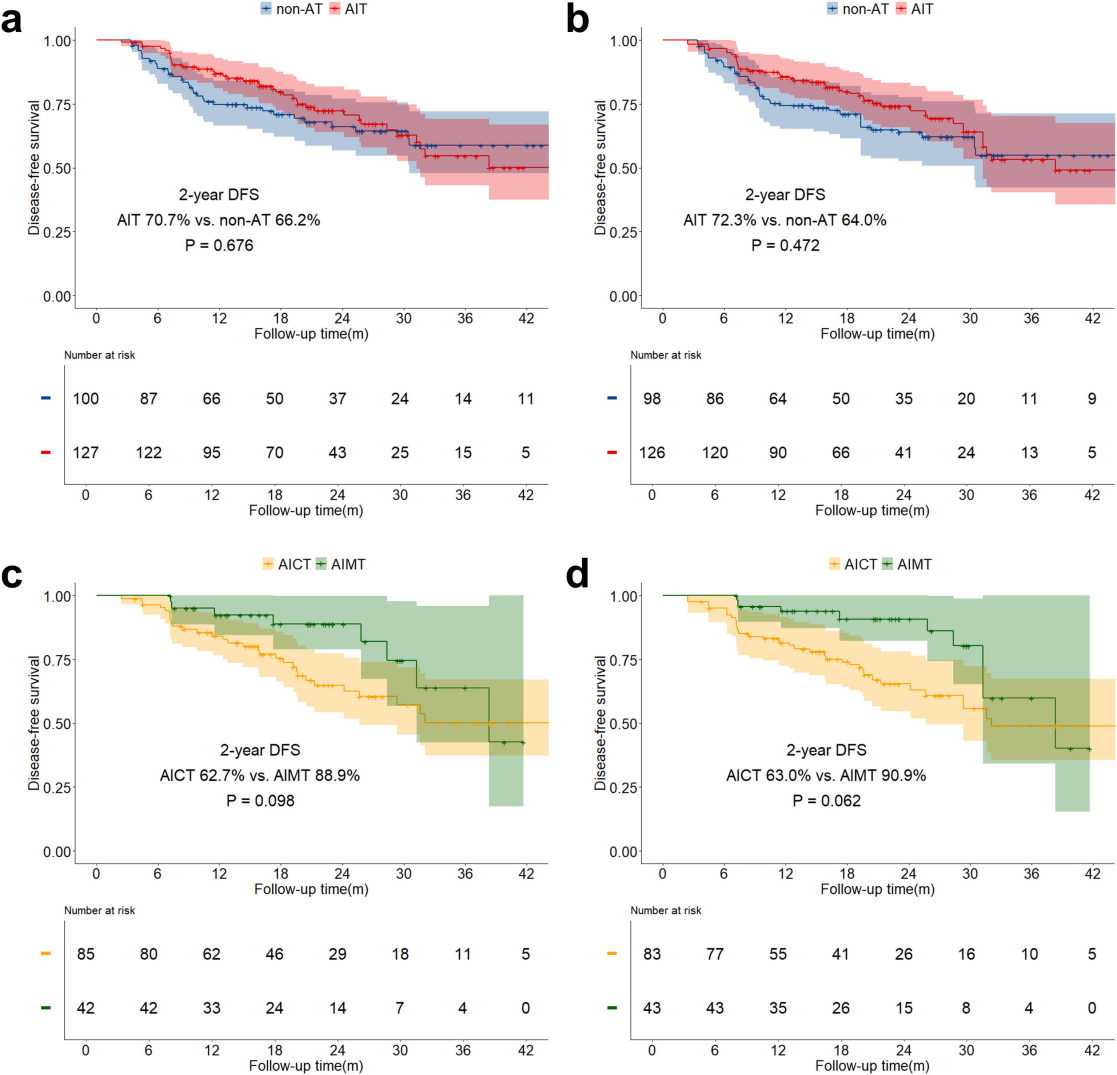
DFS curves before and after sIPTW for non-pCR patients, stratified by adjuvant therapy status. **(a)** Comparison of DFS between the AIT group and the non-AT group before sIPTW; **(b)** Comparison of DFS between the AIT group and the non-AT group after sIPTW; **(c)** Comparison of DFS between the AICT group and the AIMT group before sIPTW; **(d)** Comparison of DFS between the AICT group and the AIMT group after sIPTW.

**Figure 10:**
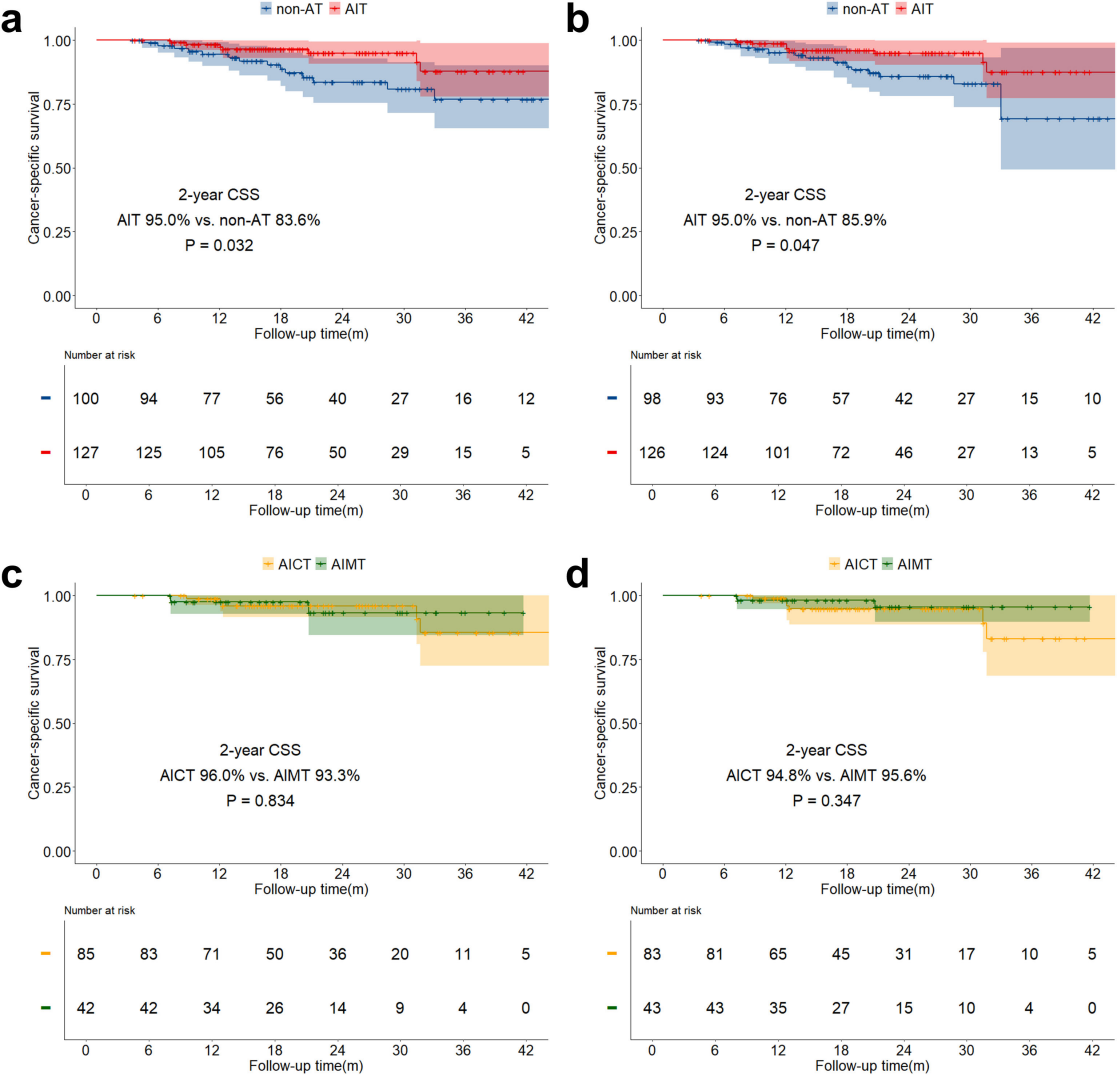
CSS curves before and after sIPTW for non-pCR patients, stratified by adjuvant therapy status. **(a)** Comparison of CSS between the AIT group and the non-AT group before sIPTW; **(b)** Comparison of CSS between the AIT group and the non-AT group after sIPTW; **(c)** Comparison of CSS between the AICT group and the AIMT group before sIPTW; **(d)** Comparison of CSS between the AICT group and the AIMT group after sIPTW.

In the stratified analysis of pCR patients, neither OS, DFS, nor CSS in the AIT group showed statistically significant benefits compared to the non-AT group before and after sIPTW (**Figures 5a, b**; **Figures 6a, b**; **Figures 7a, b**). However, it was worth noting that no deaths were observed in the AIT group. The AICT group and the AIMT group did not show any statistical differences in OS, DFS, or CSS neither (**Figures 5c, d**; **Figures 6c, d**; **Figures 7c, d**). In the stratified analysis of non-pCR patients, both before and after sIPTW, the OS and CSS in the AIT group was significantly better than that in the non-AT group (**Figures 8a, b**; **Figures 9a, b**). There were no significant differences in DFS between the two groups (**Figures 10a, b**). No statistical differences were found in OS, DFS, or CSS between the AICT and AIMT groups (**Figures 8c, d**; **Figures 9c, d**; **Figures 10c, d**). Further comparison of AICT and AIMT in ypN0 (no lymph node residual) and ypN+ (positive lymph nodes) within non-pCR patients showed no significant differences in survival outcomes except DFS in ypN+ after sIPTW (AICT vs. AIMT: 35.8% vs. 85.8%, P = 0.016) (**Supplementary Figure 1**-**3**).

### Patterns of recurrence and metastasis

During the follow-up period, a total of 66 patients (20.4%) experienced recurrence or metastasis. Among them, 36 patients (22.1%) in the AIT group and 30 patients (18.7%) in the non-AT group developed recurrence. The specific patterns of recurrence and metastasis for both groups are detailed in **Table 4**. No statistically significant differences were observed in the patterns of recurrence and metastasis between the two groups. Within the pCR subgroup, 2 patients in the AIT group (5.6%) developed recurrence or metastasis, with one regional lymph node recurrence and one liver metastasis; 5 patients (8.3%) in the non-AT group developed recurrence or metastasis. No statistically significant differences were observed between the two groups within the pCR subgroup (**Supplementary Table 2**).

**Table 4:** Patterns of recurrence and metastasis.

Patterns of recurrence and metastasis	AIT group (n=163)	Non-AT group(n=160)	P
No recurrence	127 (77.9)	130 (81.3)	0.83
Regional recurrence	19 (11.7)	14 (8.8)	
Distant metastasis	12 (7.4)	11 (6.9)	
Concurrent	5 (3.1)	5 (3.1)	

## Discussion

Since the CROSS study (7), NEOCRTEC5010 study (8), and JCOG9907 study (9) demonstrated significant survival benefits of neoadjuvant therapy for patients with LA-ESCC, nCRT or neoadjuvant chemotherapy (nCT) has become the standard treatment. However, the necessity of adjuvant therapy after neoadjuvant treatment remains controversial. However, the Checkmate-577 study (2) showed that adjuvant nivolumab could double the median DFS in patients with residual disease after nCRT. Based on this study, guidelines in several countries have incorporated postoperative AIT as a first-line recommendation for ESCC patients with residual disease after neoadjuvant treatment. With the further advancement of immunotherapy in the treatment of ESCC, nICT has become the predominant treatment strategy for LA-ESCC in China. Compared to nCT, the addition of immunotherapy has significantly improved pathological response rates. Interim results from the HCHTOG1909 study (6) also indicate that nICT can improve prognosis. However, the need for adjuvant therapy after nICT for locally advanced ESCC remains debated. Current phase III clinical trials predominantly adopt the nICT-surgery-AIT model, lacking head-to-head comparisons between neoadjuvant therapy and perioperative therapy (5, 6).

Previous studies have yielded inconsistent conclusions regarding the benefits of adjuvant therapy after neoadjuvant treatment. A single-arm study by Nomura et al. (10) showed that adjuvant S-1 chemotherapy after nCT resulted in higher RFS and OS (3-year RFS: 72.3%, 3-year OS: 85.0%; compared to the JCOG9907 study, 3-year RFS: 49.2%, 3-year OS: 62.7%). A meta-analysis by Lee et al. (1) also demonstrated that adjuvant therapy after nCRT or nCT could improve OS. In contrast, Xie et al. (11) found that adjuvant chemotherapy after nICT not only failed to improve prognosis but also compromised OS and RFS, and reported that AICT after nICT did not improve patient prognosis either. A meta-analysis (12) of immunotherapy for non-small cell lung cancer showed no survival difference between neoadjuvant therapy and perioperative therapy, suggesting that adjuvant immunotherapy after nICT might be unnecessary. This study found that adjuvant immunotherapy after nICT did not improve DFS but significantly improved OS and CSS. This suggests that while postoperative adjuvant immunotherapy did not reduce the incidence of recurrence or metastasis, it improved patient survival. Several factors might explain the inconsistent findings. For example, in Xie et al.’s study, the majority of patients received only 2 cycles of nICT (11), whereas a higher proportion of patients in our cohort received 3–4 cycles. More cycles of nICT might lead to a more profound modulation of the tumor-immune microenvironment, potentially influencing the efficacy of AIT. Another critical factor was the adjuvant regimen, as in Xie et al.’s study, many patients received adjuvant chemotherapy, while in this study these patients were excluded.

In the era of neoadjuvant therapy, pCR and major pathological response (MPR) are considered potential surrogate endpoints for patient survival. In esophageal cancer, a study by Hong et al. (13) demonstrated that MPR could serve as a surrogate endpoint for survival in patients receiving neoadjuvant chemotherapy or neoadjuvant immunochemotherapy. There have been some studies highlighting the importance of giving adjuvant therapy based on the pCR or MPR status. In a large-scale multicenter study, Chen et al. reported that AIT provided no survival benefit in the overall nICT ESCC cohort (OS: HR = 0.87, P = 0.12; DFS: HR = 1.03, P = 0.69) but improved OS in non-pCR subgroup (HR = 0.72, P = 0.031) and non-MPR subgroup (HR = 0.56, P = 0.035) (14). Liu et al.’s study also reported non-pCR patients benefited from adjuvant therapy in DFS (P = 0.042) and OS (P = 0.033) (15). Feng et al. (16) reported that AIT after nICT improved DFS (3-year DFS: 23.9% vs. 38.5%, P = 0.036) and OS (3-year OS: 37.0% vs. 61.5%, P = 0.010) in ypT+N+ patients, but no significant statistical differences were observed in ypT0N0 patients. Hong et al. (13) found that for ESCC patients achieving MPR after nICT or nCT, AT did not affect prognosis. Conversely, a study by Zhao et al. (17) on non-small cell lung cancer showed that postoperative adjuvant immunotherapy did not improve survival in patients who achieved pCR or MPR after neoadjuvant immunochemotherapy. In Xie et al.’s study (11), subgroup analysis based on pCR status revealed that neither adjuvant chemotherapy nor AICT improved prognosis in either pCR or non-pCR patients. In this study, we conducted stratified analyses based on whether patients achieved pCR. Our results showed that within the current sample size and follow-up period, no significant survival benefit from AIT was observed for pCR patients. However, in the non-pCR population, although AIT did not improve DFS, it did confer benefits in OS and CSS. This conclusion aligns with the majority of current opinions, where it is generally believed that patients with residual tumors derive greater benefits from postoperative adjuvant therapy, while pCR populations experience limited benefits. In current clinical practice, pCR patients are generally managed with observation, while non-pCR patients are often recommended AIT based on the findings of the Checkmate-577 study. Our results support the current mainstream approach, suggesting that AIT can improve prognosis in non-pCR patients. However, although there was no significant survival benefit from AIT for pCR patients, it should be noticed that pCR patients receiving AIT seemed to have better survival outcomes (no death within the current follow-up period). The statistical power might be constrained by limited sample size and relatively short follow-up period. Consequently, clinical decision for pCR patients awaits validation in larger cohorts with extended follow-up.

A confusing question in the study was the inconsistency between DFS and OS/CSS, which was also reported by Chen et al. (14). The inconsistency between DFS and OS/CSS may be attributed to several factors: First, nICT has already significantly reduced the rate of postoperative recurrence and metastasis. The tumor cells in the remaining micro-metastatic foci may have developed resistance, meaning that AIT cannot completely eliminate these micro-metastases. However, ICIs could reprogram the tumor-immune microenvironment, strengthen immune response, and offer potential long-term benefits (known as “long-tail effect) (18, 19). Second, there may be a selection bias among patients, as those receiving AIT inherently have a higher risk of recurrence and metastasis. Therefore, this subgroup of patients was at a greater baseline risk of recurrence, but the improvement was not reflected in the study results. Third, the subsequent therapy might influence the OS for patients. However, subsequent therapy data was absent in this retrospective study, making the analysis unavailable. Fourth, another possible bias is that patients receiving AIT may have better overall health status and longer expected lifespans. Due to the retrospective and observational nature of this study, we cannot further validate these hypotheses, and the conclusions warrant confirmation through subsequent high-quality meta-analyses and large randomized controlled trials.

Another interesting result in this study was that the AICT group had significantly poorer DFS than the AIMT group in ypN+ patients. This might be due to insufficient neoadjuvant cycles (3–4 cycles between AICT and AIMT after sIPTW: 63.9% vs. 89.3%, P = 0.029, **Supplementary Table 3**) and heavier residual tumor burden (IPR between AICT and AIMT after sIPTW: 83.3% vs. 50.5, P = 0.012; **Supplementary Table 3**). While poorer DFS was observed in the AIMT group, the OS and CSS were comparable to the AICT group, which might be partly explained by the reasons mentioned above. However, as the sIPTW method was exclusively applied in the comparison between AIT and non-AT, the direct comparison between AICT and AIMT remained unadjusted. Consequently, a cautious interpretation of this finding should be warranted.

Postoperative recurrence and metastasis remain critical challenges in ESCC management. Long-term follow-up results from the CROSS, NEOCRTEC5010, and CMISG1701 studies (7, 8, 20) showed that 30%-50% of patients experienced recurrence or metastasis after nCRT, predominantly distant metastasis. In this study, the recurrence and metastasis rate in ESCC patients after nICT was significantly lower (20.5%), reflecting the improved prognosis associated with the nICT treatment model. However, the pattern of recurrence and metastasis still predominantly involved distant sites (33 cases, 50%). Additionally, there were no significant differences in the incidence or patterns of recurrence and metastasis between the adjuvant therapy and non-adjuvant therapy groups, suggesting that adjuvant immunotherapy does not alter the recurrence rate or patterns in ESCC patients postoperatively.

This study has several limitations. First of all, as a retrospective study, it was inherently susceptible to selection bias and information bias even employing the sIPTW method to adjust known confounders. Unmeasured confounding factors (e.g., patient performance status, comorbidities, specific differences in immunotherapy regimens, treatment adherence) cannot be controlled. Moreover, the reasons for administering adjuvant treatment and the specific regimens used were not clearly defined. Secondly, this study did not include the biomarker analysis (especially PD-1/PD-L1 status, which showed a significant role in the *post-hoc* analysis of Checkmate-577 (21)), limiting the ability to compare subgroups stratified by biomarker expression status. Thirdly, the lack of safety data, particularly regarding immune-related adverse events (irAEs), was an important limitation. To avoid the significant bias that would arise from analyzing highly incomplete safety data, we did not include a formal safety analysis. However, future prospective studies must rigorously document safety profiles, because safety is a crucial factor in deciding treatment regimens. Fourthly, the median follow-up time in this study was 21.6 months, which was relatively limited.

In conclusion, we found that AIT improves prognosis in LA-ESCC patients following nICT and R0 resection, especially for those non-pCR patients.

## Data Availability

The raw data supporting the conclusions of this article will be made available upon reasonable request to corresponding authors.
