# Supplementary material for: Impact of adjuvant immunotherapy on prognosis in esophageal squamous cell carcinoma patients following neoadjuvant immunochemotherapy
Source: Front Oncol. 2026 Mar 18;16:1735049. doi: 10.3389/fonc.2026.1735049 (PMC13058475; doi:10.3389/fonc.2026.1735049)
Supplement: Supplementary file 1 [file DataSheet1.docx]

Supplementary Material

# Supplementary Table 1 Regimens of adjuvant therapy

| Adjuvant therapy | Number (n, %) |
| --- | --- |
| Adjuvant regimen (n = 163) |  |
| AICT | 108 (66·3) |
| AIMT | 55 (33·7) |
| Adjuvant chemotherapy regimen (n = 108) |  |
| TP | 100 (92·6) |
| 5-Fu/S-1 | 7 (6·5) |
| TP+5-Fu/S-1 | 1 (0·9) |
| ICI regimen (n = 163) |  |
| PD-1 | 162 (99·4) |
| PD-L1 | 1 (0·6) |

**Supplementary Table 2 Recurrence and metastasis patterns between the AIT and non-AT groups within pCR patients**

| Patterns of Recurrence and Metastasis | AIT (n=36) | Non-AT (n=60) | P |
| --- | --- | --- | --- |
| No recurrence | 34 (94.4) | 55 (91.7) | 0.61 |
| Regional recurrence | 1 (2.8)^a^ | 1 (1.7) |  |
| Distant metastasis | 1 (2.8)^b^ | 1 (1.7) |  |
| Concurrent | 0 | 3 (5.0) |  |

1. A patient had regional lymph node recurrence after immune checkpoint inhibitor monotherapy (AIMT).
2. A patient had liver metastasis after adjuvant immunochemotherapy (AICT).

**Supplementary Table 3 Baseline characteristics and pathologic outcomes between the AICT group and the AIMT group within ypN+ patients**

| Characteristic | Before sIPTW | | | | After sIPTW | | | |
| --- | --- | --- | --- | --- | --- | --- | --- | --- |
|  | **AICT** | **AIMT** | **SMD** | **P** | **AICT** | **AIMT** | **SMD** | **P** |
| Number | 35 | 21 |  |  | 31 | 19 | 0.914 | 0.003 |
| Sex (n, %) |  |  | 0.862 | 0.006 |  |  |  |  |
| Male | 34 (97.1) | 14 (66.7) |  |  | 29.8 (96.8) | 12.2 (63.7) |  |  |
| Female | 1 (2.9) | 7 (33.3) |  |  | 1.0 (3.2) | 6.9 (36.3) |  |  |
| Age (n, %) |  |  | 0.213 | 0.623 |  |  | 0.309 | 0.291 |
| ≥65 years | 13 (37.1) | 10 (47.6) |  |  | 11.7 (37.9) | 10.2 (53.1) |  |  |
| <65 years | 22 (62.9) | 11 (52.4) |  |  | 19.1 (62.1) | 9.0 (46.9) |  |  |
| Smoking history (n, %) | 19 (54.3) | 14 (66.7) | 0.255 | 0.528 | 17.9 (58.2) | 12.2 (63.7) | 0.111 | 0.706 |
| Drinking history (n, %) | 12 (34.4) | 9 (42.9) | 0.177 | 0.722 | 11.2 (36.4) | 8.3 (43.4) | 0.142 | 0.627 |
| Location (n, %) |  |  | 0.412 | 0.361 |  |  | 0.449 | 0.241 |
| Upper | 3 (8.6) | 1 (4.8) |  |  | 1.7 (5.7) | 0.6 (3.2) |  |  |
| Middle | 10 (28.6) | 3 (14.3) |  |  | 8.7 (28.3) | 2.3 (12.0) |  |  |
| Lower | 22 (62.9) | 17 (81.0) |  |  | 20.3 (66.0) | 16.2 (84.8) |  |  |
| Clinical stage (n, %) |  |  | 0.348 | 0.439 |  |  | 0.339 | 0.446 |
| II | 7 (20.0) | 5 (23.8) |  |  | 6.9 (22.4) | 5.2 (26.9) |  |  |
| III | 25 (71.4) | 12 (57.1) |  |  | 22.1 (71.7) | 11.1 (58.3) |  |  |
| IV | 3 (8.6) | 4 (19.0) |  |  | 1.8 (5.9) | 2.8 (14.8) |  |  |
| Neoadjuvant chemotherapy regimen (n, %) |  |  | 0.753 | 0.254 |  |  | 0.277 | 0.244 |
| TP/DP | 34 (97.1) | 19 (90.5) |  |  | 30.1 (97.8) | 17.5 (91.7) |  |  |
| CF | 1 (2.9) | 2 (9.5) |  |  | 0.7 (2.2) | 1.6 (8.3) |  |  |
| Neoadjuvant ICIs (n, %) |  |  | 0.100 | >0.99 |  |  | 0.037 | 0.898 |
| PD-1 | 34 (97.1) | 20 (95.2) |  |  | 29.6 (96.3) | 18.3 (95.5) |  |  |
| PD-L1 | 1 (2.9) | 1 (4.8) |  |  | 1.2 (3.7) | 0.9 (4.5) |  |  |
| Neoadjuvant cycles (n, %) |  |  | 0.604 | 0.084 |  |  | 0.629 | 0.029 |
| 1-2 | 14 (40.0) | 3 (14.3) |  |  | 11.1 (36.1) | 2.0 (10.7) |  |  |
| 3-4 | 21 (60.0) | 18 (85.7) |  |  | 19.7 (63.9) | 17.1 (89.3) |  |  |
| ypT (n, %) |  |  | 0.753 | 0.254 |  |  | 0.931 | 0.048 |
| T0 | 2 (5.7) | 5 (23.8) |  |  | 1.4 (4.6) | 6.5 (33.8) |  |  |
| Tis | 2 (5.7) | 1 (4.8) |  |  | 1.2 (3.9) | 0.7 (3.4) |  |  |
| T1 | 6 (17.1) | 5 (23.8) |  |  | 5.1 (16.6) | 3.9 (20.5) |  |  |
| T2 | 7 (20.0) | 1 (4.8) |  |  | 6.2 (20.1) | 1.1 (5.6) |  |  |
| T3 | 17 (48.6) | 9 (42.9) |  |  | 16.3 (53.0) | 7.0 (36.6) |  |  |
| T4 | 1 (2.9) | 0 (0.0) |  |  | 0.6 (1.8) | 0.0 (0.0) |  |  |
| ypN (n, %) |  |  | 0.537 | 0.316 |  |  | 0.707 | 0.034 |
| N1 | 25 (71.4) | 19 (90.5) |  |  | 20.4 (66.3) | 17.7 (92.4) |  |  |
| N2 | 8 (22.9) | 2 (9.5) |  |  | 7.9 (25.6) | 1.4 (7.6) |  |  |
| N3 | 2 (5.7) | 0 (0.0) |  |  | 2.5 (8.1) | 0.0 (0.0) |  |  |
| ypTNM (n, %) |  |  | 0.348 | 0.523 |  |  | 0.420 | 0.515 |
| I | 0 (0.0) | 0 (0.0) |  |  | 0 (0.0) | 0 (0.0) |  |  |
| II | 0 (0.0) | 0 (0.0) |  |  | 0 (0.0) | 0 (0.0) |  |  |
| III | 33 (94.3) | 21 (100.0) |  |  | 28.3 (91.9) | 19.1 (100) |  |  |
| IV | 2 (5.7) | 0 (0.0) |  |  | 2.5 (8.1) | 0 (0.0) |  |  |
| Pathological response (n, %) |  |  | 0.508 | 0.127 |  |  | 0.744 | 0.012 |
| MPR | 7 (20.0) | 9 (42.9) |  |  | 5.1 (16.7) | 9.5 (49.5) |  |  |
| IPR | 28 (80.0) | 12 (57.1) |  |  | 25.6 (83.3) | 9.7 (50.5) |  |  |


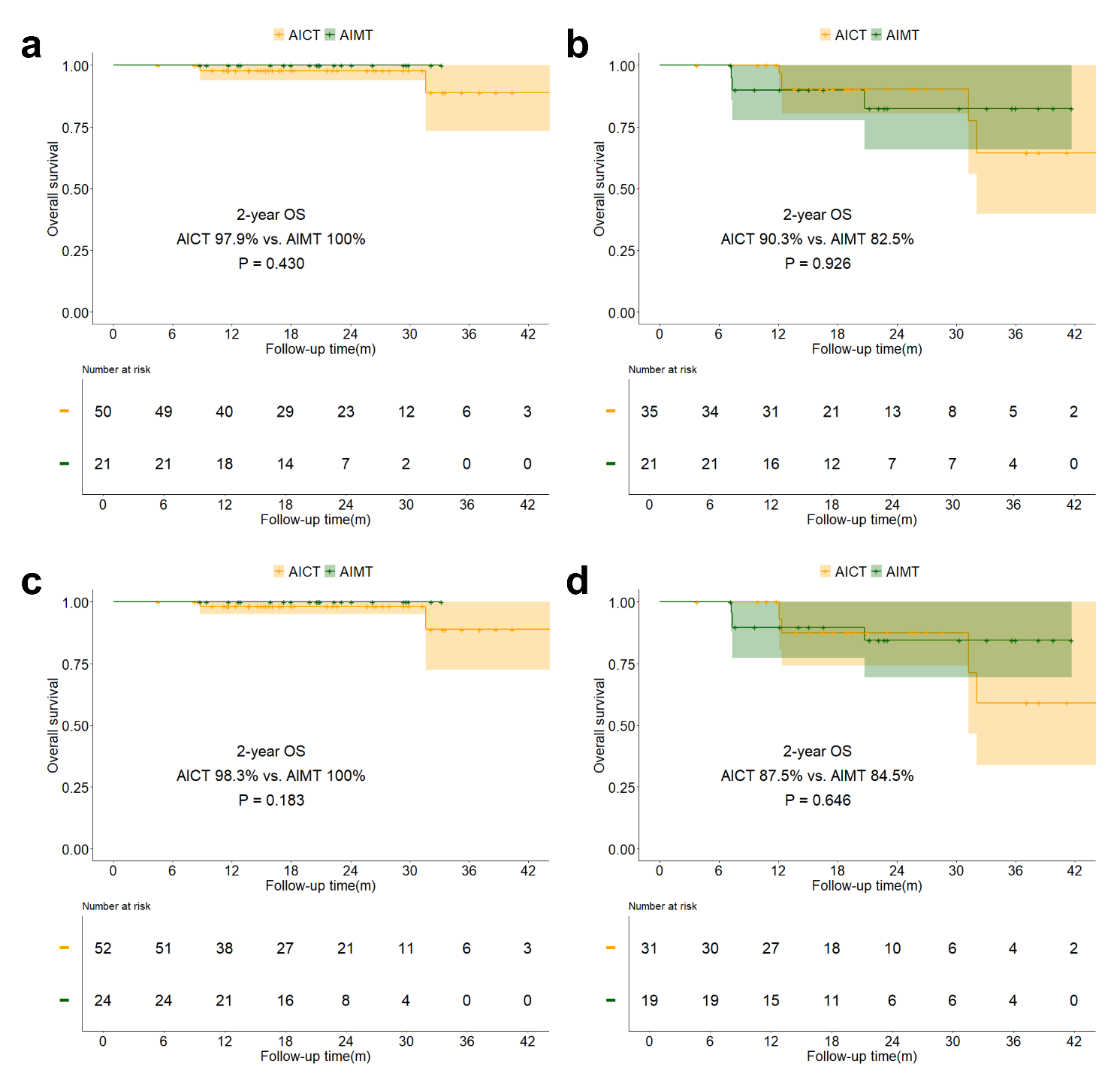


**Supplementary Figure 1· OS curves between the AICT group and the AIMT group before and after sIPTW in non-pCR patients, stratified by ypN0 and ypN+.** a. Comparison of OS between the AICT group and the AIMT group within ypN0 before sIPTW; b. Comparison of OS between the AICT group and the AIMT group within ypN+ before sIPTW; c. Comparison of OS between the AICT group and the AIMT group within ypN0 after sIPTW; d. Comparison of OS between the AICT group and the AIMT group within ypN+ after sIPTW.


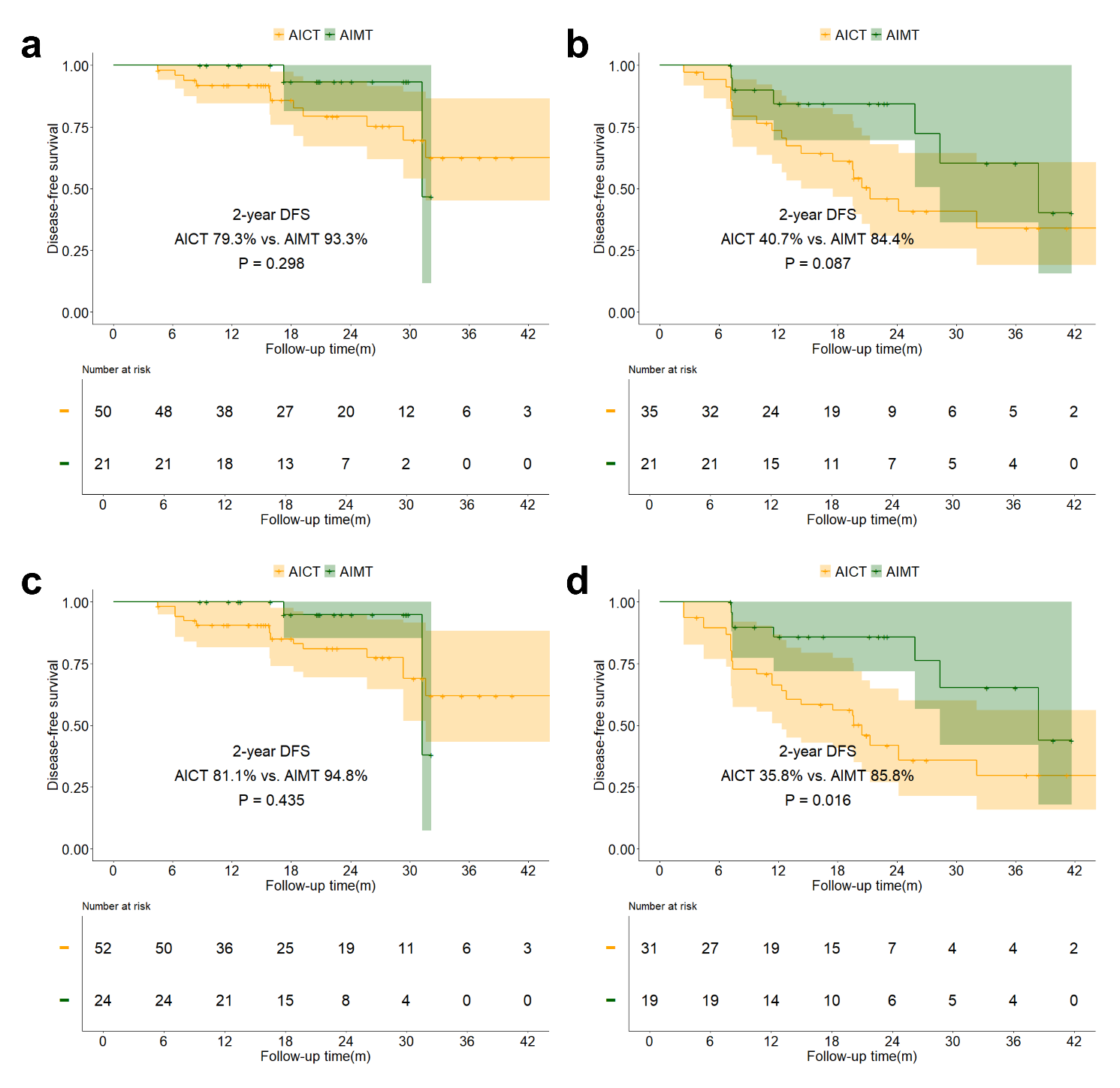


**Supplementary Figure 2· DFS curves between the AICT group and the AIMT group before and after sIPTW in non-pCR patients, stratified by ypN0 and ypN+.** a. Comparison of DFS between the AICT group and the AIMT group within ypN0 before sIPTW; b. Comparison of DFS between the AICT group and the AIMT group within ypN+ before sIPTW; c. Comparison of DFS between the AICT group and the AIMT group within ypN0 after sIPTW; d. Comparison of DFS between the AICT group and the AIMT group within ypN+ after sIPTW.


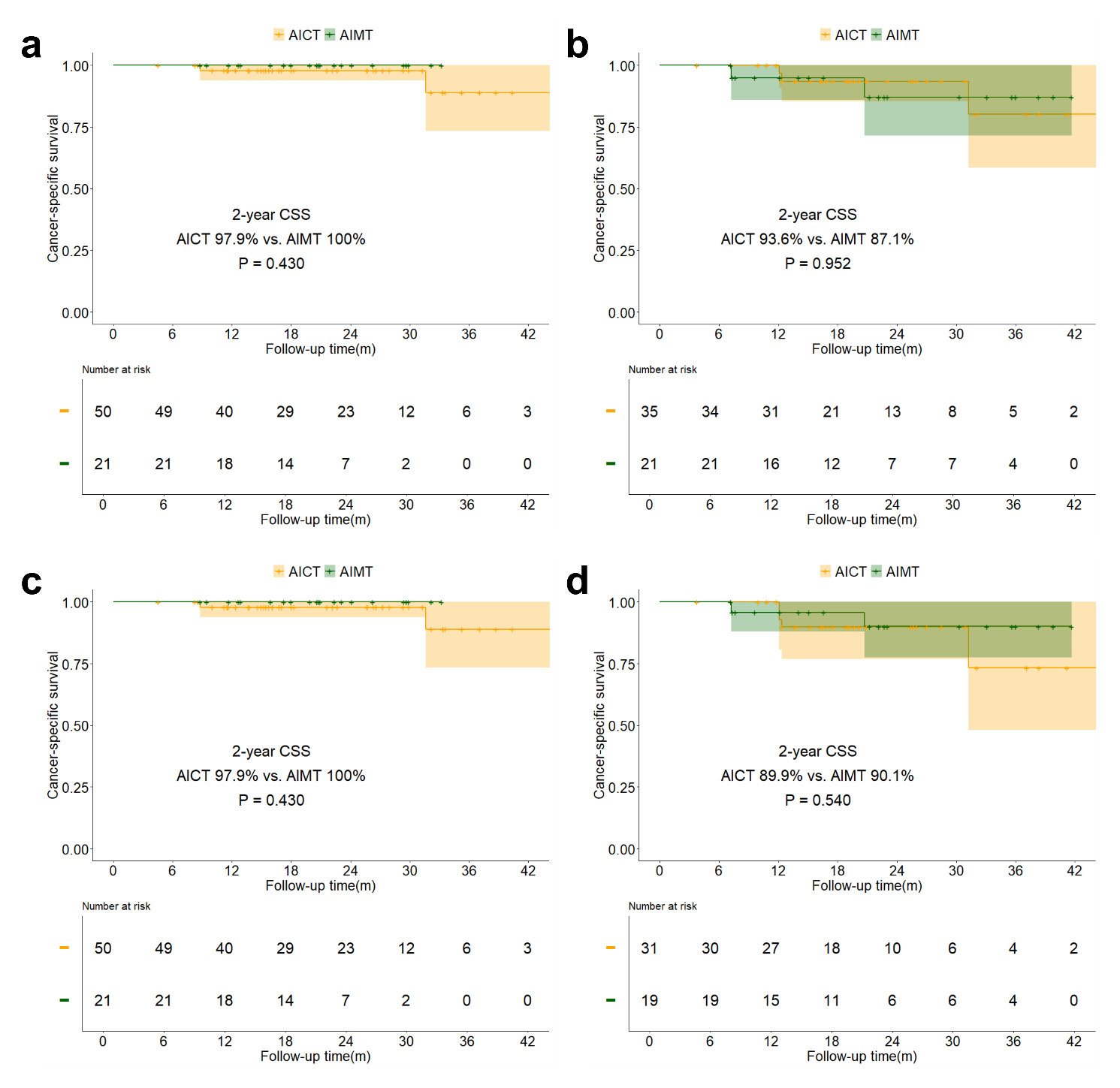


**Supplementary Figure 3· CSS curves between the AICT group and the AIMT group before and after sIPTW in non-pCR patients, stratified by ypN0 and ypN+.** a. Comparison of CSS between the AICT group and the AIMT group within ypN0 before sIPTW; b. Comparison of CSS between the AICT group and the AIMT group within ypN+ before sIPTW; c. Comparison of CSS between the AICT group and the AIMT group within ypN0 after sIPTW; d. Comparison of CSS between the AICT group and the AIMT group within ypN+ after sIPTW.
